# Clinical and Imaging Features of Primary Cardiac Angiosarcoma

**DOI:** 10.3390/diagnostics10100776

**Published:** 2020-09-30

**Authors:** Yan Chen, Yu Li, Nan Zhang, Jianfeng Shang, Xiaodan Li, Jiayi Liu, Lei Xu, Dongting Liu, Zhonghua Sun, Zhaoying Wen

**Affiliations:** 1 Department of Radiology, Beijing Anzhen Hospital, Capital Medical University, Chaoyang District, Beijing 100029, China; chenyanjlu2013@163.com (Y.C.); athen06@hotmail.com (Y.L.); nzhang_1987@163.com (N.Z.); lxdcpyyxf@163.com (X.L.); ljy76519@163.com (J.L.); leixu2001@hotmail.com (L.X.); dongting0530@163.com (D.L.); 2 Department of Pathology, Beijing Anzhen Hospital, Capital Medical University, Chaoyang District, Beijing 100029, China; azshangjianfeng@163.com; 3 Discipline of Medical Radiation Sciences, School of Molecular and Life Sciences, Curtin University, Perth 6845, Australia

**Keywords:** heart, angiosarcoma, computed tomography, magnetic resonance imaging

## Abstract

This study aims to explore computed tomography (CT) and magnetic resonance imaging (MRI) features of patients diagnosed with primary cardiac angiosarcoma. The study involved the analysis of 12 patients diagnosed with primary cardiac angiosarcoma who underwent non-contrast (8/12) or contrast-enhanced CT (10/12) or MRI (4/12). Imaging appearances, including the tumor location and adjacent infiltration, were analyzed. All 12 lesions were located in the right atrium with a broad base. Adjacent invasion including the tricuspid valve and right ventricle (2/12), inferior or superior vena cava (2/12), pericardium (10/12), and right coronary artery (7/12) was common. On unenhanced CT scans, tumors in two patients were homogeneous in density, whereas the others were inhomogeneous. Ten patients showed heterogeneous enhancement. The enhancement pattern showed no direct correlation with the differentiation degree of the tumor. Four lesions manifested as heterogeneous intensity, with hyperintense hemorrhage foci on both T1- and T2-weighted MRI. Three showed rim enhancement and one showed patchy enhancement. Primary cardiac angiosarcoma often involves the right side of the heart with infiltration of peripheral structures. CT features include typical inhomogeneous density on unenhanced scans and heterogeneous centripetal enhancement on enhanced scans. A cauliflower-like appearance on both T1- and T2-weighted MRI is common. The characteristic enhancement pattern of MRI remains to be determined.

## 1. Introduction

Soft tissue sarcoma is a rare malignancy originating from mesenchymal cells, with a prevalence of <1% of all adult cancers [1]. Primary cardiac sarcoma, which is an extremely rare subtype of soft tissue sarcoma, has an incidence at autopsy of approximately 0.001–0.03% and a general incidence of 0.007% [2]. Primary cardiac angiosarcoma is the most common subset, accounting for approximately 30% of those cases [3]. Primary cardiac angiosarcoma predominantly occurs in men with a male/female ratio of 2–3/1 [4], and it may occur at any age and anywhere in the heart; however, people aged between 30 and 50 years, and the right atrium, are the most frequently affected [5]. Clinical manifestations of primary cardiac angiosarcomas are not specific and depend on their infiltration into the myocardium and adjacent structures as well as the extent of metastases [6]. Patients usually visit a hospital for treatment because of their complications including chest pain, arrhythmia, peripheral edema, dyspnea, orthopnea, congestive heart failure, and pericardial tamponade [7]. The prognosis of primary cardiac angiosarcoma is considerably poor owing to many reasons including late diagnosis, extensive metastases, incomplete resection, unsatisfactory effect of adjuvant therapy, and lack of standard treatment. According to recent case series [8,9,10,11], the median overall survival ranges from several days to several months. The diagnosis is challenging and usually remains exclusive. The use of noninvasive imaging modalities for a comprehensive analysis of tumor features can improve the early detection of cardiac neoplasms and may provide a specific classification before histopathological analysis [6]. However, the imaging characteristics of primary cardiac angiosarcoma are not well understood because most of the current knowledge is mainly based on case reports. Therefore, we collected the clinical, computed tomography (CT), and magnetic resonance imaging (MRI) data of 12 cases and analyzed the imaging characteristics of primary cardiac angiosarcoma to improve understanding of the imaging features of this particular disease, hence achieving the goal of earlier diagnosis and better prognosis.

## 2. Methods

### 2.1. Clinical Data

This retrospective study reviewed and analyzed 12 cases of pathologically confirmed primary cardiac angiosarcoma from June 2009 to November 2019. Of these patients, 8 were from our hospital (Beijing Anzhen Hospital, Capital Medical University) and the remaining 4 were from different medical institutions (including Shanghai Dongfang Hospital, Shengjing Hospital of China Medical University, Cangzhou Capital Hospital and Henan Provincial Chest Hospital). This study was approved by the hospital Ethics Committee who waived the requirement for written informed consent because patients’ details were anonymous.

In total, there were 10 men and 2 women, with an average age of approximately 45 ± 18 (range: 17–74) years. The median time from onset to treatment was 1 (range: 0.5–12) month. Clinical manifestations were not specific; the most common complaints included exertional dyspnea, palpitation, and chest pain or hemoptysis. Two patients had a history of tuberculosis, and none of them had familial or other specific histories. Laboratory test results showed an elevated serum carbohydrate antigen-125 (CA-125) level in three patients, of whom two were men.

### 2.2. Imaging Data

Unenhanced and/or contrast-enhanced CT images were available for all the patients; however, MRI data were available only for four patients.

The CT images were acquired using different scanners including 2 × 128-slice CT (Somatom Definition Flash, Siemens Healthcare, Forchheim, Germany), 256-slice CT (Revolution CT, GE Healthcare, Milwaukee, WI, USA), and 320-slice CT (Aquilion One, Toshiba, Otawara, Japan). The tube voltage was 100 or 120 kV as automatically determined by the kV assist; the tube current was set 300 mA with dynamic current modulation; matrix size was 512 × 512, collimation was 256 × 0.6/0.625 mm or 320 × 0.5 mm, and reconstruction slice thickness was 1 mm. All the patients who underwent enhanced CT examination were intravenously injected with a contrast agent (370 mg of iodine/mL, Ultravist, Bayer Schering Pharma, Berlin, Germany) and 30 mL saline solution with a scan delay of approximately 25 s for the arterial phase and 60 s for the venous phase after the onset of injection. The flow volume was 50–60 mL, and the flow rate was 3.0 mL/s.

The MRI images were obtained using 3.0 T (Magnetom Verio, Siemens, Erlangen, Germany) and 3.0 T (Discovery MR750w, GE healthcare). Heart coils were used. All the patients were scanned using routine protocols. T1- and T2-weighted black blood images were acquired in the axial plane, followed by fat-suppressed images. Short axis, 2-chamber, 4-chamber, and left ventricular outflow tract cines were obtained for the functional assessment. Short axis and/or axial rest perfusion stacks through the center of the mass were obtained using gadopentetate di-meglumine (Gd-DTPA). After approximately 10 min, late-phase gadolinium enhancement (LGE) images in short axis, 2-chamber, and 4-chamber planes through the center of the mass were acquired. Gd-DTPA was injected at a rate of 1 mL/s, with a dose of 0.1 mmol/kg for LGE.

PET-CT (Siemens Biograph 16, Siemens Medical Solutions) examination was performed in 4 patients.

### 2.3. Image Analysis

All the imaging data were evaluated by three specialists who had more than 15 years of experience in diagnostic imaging of the cardiovascular system. Tumor features analyzed were as follows: location, shape, maximum size, density/intensity, margin, infiltration, and enhancement pattern.

### 2.4. Pathological Analysis

The samples of all the patients were conventionally embedded, fixed, and stained with hematoxylin and eosin and typed through immunohistochemistry. Three experienced pathologists majoring in cardiovascular diagnosis read the results.

## 3. Results

### 3.1. General Results and Follow-Up

Clinical and imaging characteristics are listed in Table 1. Overall, six patients underwent radical surgical operation and were discharged with stable condition; two died six and seven months, respectively, after resection; one is still alive (with a survival of more than 15 months); and three were lost to follow-up. In two patients who underwent thoracotomy, it was found that the lesion had infiltrated too extensively to be completely excised, and biopsy specimens were cut off before closure. One of them had aggressive progression and had a relapse approximately one month later. The other patient received chemotherapy and is still alive (with a survival of approximately seven months).

Of the four patients who could not undergo surgical treatment because of extensive metastases, two received chemotherapy, medication such as cisplatin, cyclophosphamide, paclitaxel, and vincristine was applied, one died six months after treatment, and the remaining patient is still alive (with a survival of more than nine months). Two patients were discharged without any specific treatment owing to a variety of reasons; they are still alive and undergo regular follow-ups.

### 3.2. Imaging Findings

**Figure 1:**
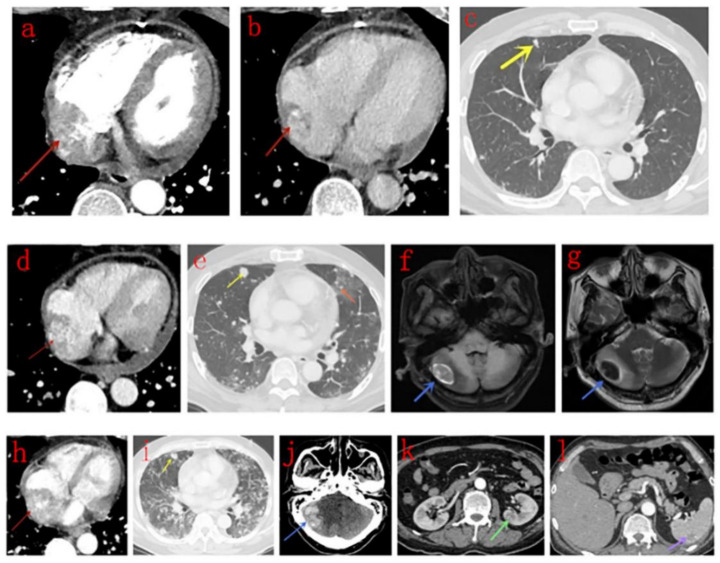
Male, 52 years old, right atrium angiosarcoma. (**a**–**c**): Contrast-enhanced computed tomography (CT) scans in the arterial (**a**) and venous (**b**) phase indicates a mass with heterogeneous centripetal enhancement (red arrow), and multiple solid nodules (**c**) are noticed in the lungs (yellow arrow). (**d**–**g**): 1.5 months later, pericardial effusion appears (**d**), metastases (yellow arrow) in the lungs are more extensive, halo sign appears (e, orange arrow). Metastasis in the right side of the cerebellum (blue arrow) presents as inhomogeneous hyperintensity on T1WI (**f**) and hypo-intensity on T2WI (**g**) with surrounding edema. (**h**–**l**): Another month later, the mass (red arrow) becomes larger, and the adjacent pericardium invasion is more extensive (**h**), and metastases in the lungs are much more extensive (**i**). The metastasis in the right side of the cerebellum (blue arrow) is larger (**j**). Metastases in the kidney (**k**, green arrow) and spleen (**l**, purple arrow) appear.

All tumors were located in the right atrium with a broad base, 11 were adhered to the free wall (10 to the lateral wall and 1 to the posterior wall), and one was attached to the atrial septum, wherein the right atrium was significantly enlarged. The pericardium adjacent to the right atrium was infiltrated in 10 cases. Two invaded the tricuspid valve and extended into the right ventricle, and two protruded into the superior or inferior vena cava. Metastases occurred in eight patients, with the lungs being the most frequently involved organ (8/12 patients, with pleural effusion in three patients), followed by the remote pericardium (3/12 patients), mediastinal lymph nodes (2/12 patients), brain (1/12 patients), kidney (1/12 patients), and spleen (1/12 patients; Figure 1). Eleven patients developed pericardial effusion, and three evolved to cardiac tamponade. The maximum size of lesions varied from 38.4 to 93.0 (range: 64.8 ± 16.6) mm.

### 3.3. CT Findings

**Figure 2:**
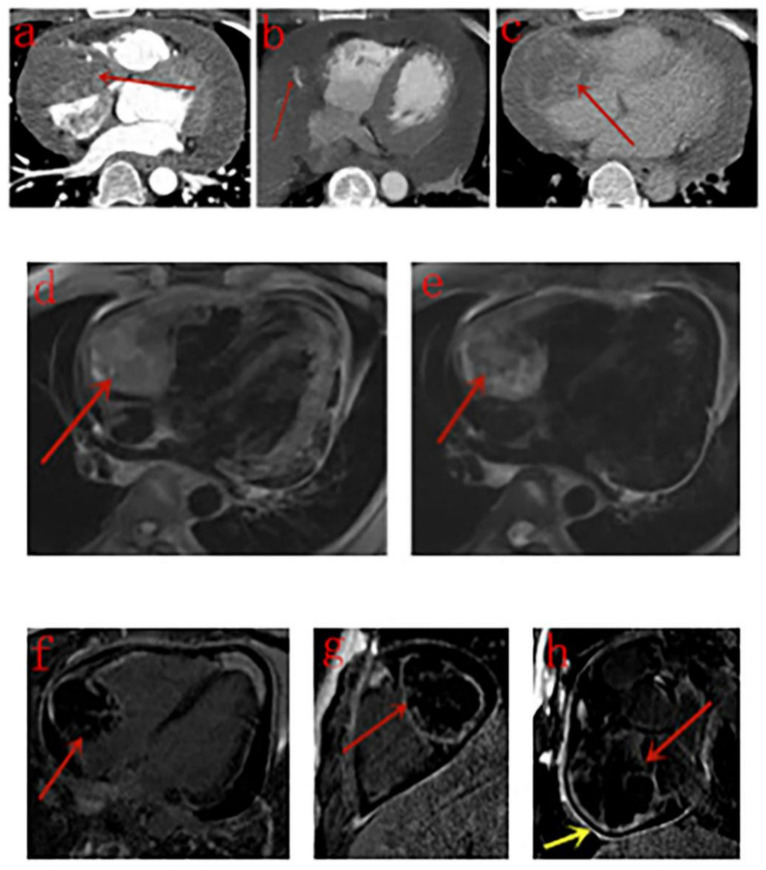
Male, 34 years old, right atrium angiosarcoma. (**a**–**c**): The mass (red arrow) is heterogeneously enhanced in the arterial (**a**) and venous (**c**) phase of contrast-enhanced CT images, and small tortuous vessels (**b**, red arrow) deriving from the right coronary artery (RCA) and pericardial effusion are noticed. (**d**,**e**): The mass (red arrow) presents as heterogeneous intensity, with multiple hyperintense hemorrhage foci on T1WI (**d**) and T2WI (**e**). (**f**–**h**): late-phase gadolinium enhancement (LGE) shows heterogeneous rim enhancement (red arrow) on four-chamber (**f**), two-chamber (**g**), and short axis (**h**) planes. Pericardium enhancement is noticed in the short axis plane (yellow arrow).

The detailed CT features of each patient are listed in Table 2. On unenhanced CT scans, lesions in two patients manifested only as an enlarged right atrium, and the other six patients showed inhomogeneous density with low-density areas. On contrast-enhanced CT scans, 10 lesions were heterogeneously enhanced in the arterial phase, and four of them showed strong enhancement. The right coronary artery (RCA) was enwrapped by the tumor in seven patients, and one of them showed small tortuous vessels deriving from the RCA to supply the neoplasm (Figure 2); in the delayed phase, the enhancement scope was broadened. Low-density areas showed no enhancement.

Upon initial diagnosis, metastatic nodules in the lungs were observed in seven patients. The nodules were surrounded by ground-glass opacity (halo sign) in five of the seven patients, and two of them also showed diffuse smooth thickening interlobular septa in both the lower lobes of the lungs. Another patient showed multiple string-of-beads effusion in the interlobular fissures of both lungs. The enhancement pattern of metastases in the remote pericardium, mediastinal lymph nodes, kidney, and spleen was the same as that of the primary lesion. Brain metastases in two patients presented as high-density areas on unenhanced CT scans.

### 3.4. MRI Findings

**Figure 3:**
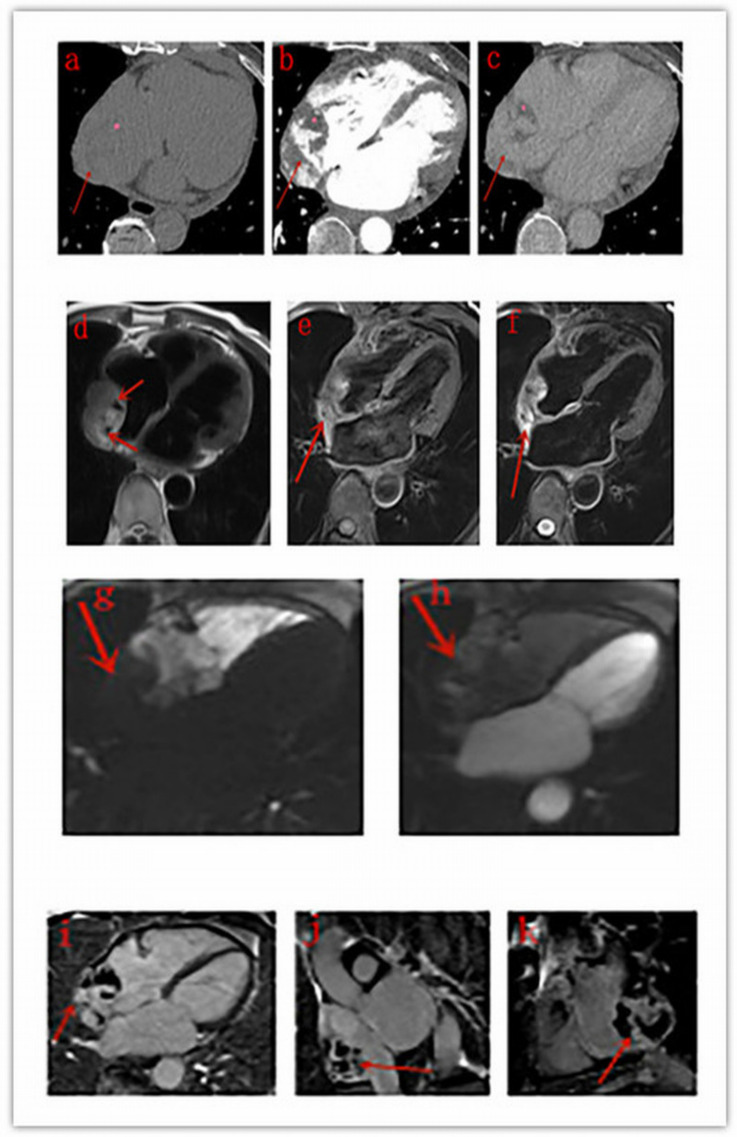
Male, 74 years old, right atrium angiosarcoma. (**a**–**c**): The mass (red arrow) in the right atrium presents as inhomogeneous density with low-density areas on plain CT scans (**a**) and shows strong heterogeneous centripetal enhancement on enhanced CT scans (**b**,**c**), and the low density area (pink star) shows no enhancement. (**d**–**f**): Signal void (red arrow) is noticed on a black-blood haste sequence (**d**). The mass (red arrow) presents as heterogeneous hyperintensity on T1WI (**e**) and T2WI (**f**). (**g**–**h**): On first-pass perfusion sequences, the mass (red arrow) presents as a filling defect in the right atrium as the contrast medium transits the right side (**g**) and is enhanced as the medium goes through the left side (**h**). (**i**–**k**): LGE shows heterogeneous patchy enhancement (red arrow) on different planes.

The detailed MRI features of each patient are listed in Table 3. Two lesions were irregular in shape, and two were approximately round. All the four lesions manifested as heterogeneous intensity, with hyperintense hemorrhage foci on both T1- and T2-weighted images compared with the myocardium. Signal void was noticed within one lesion. Cine MRI sequences showed that masses were fixed on the wall of the right atrium with a broad base and did not move with the heart; the motion of the right atrium and ventricle was impaired. First-pass perfusion sequences demonstrated a filling defect in the right atrium as the contrast medium transited the right side and enhanced masses as it went through the left side (Figure 3). Four lesions were all heterogeneously enhanced in the delayed phase, one lesion showed patchy enhancement, and the other three lesions showed rim enhancement. Pericardium enhancement was noted in one patient.

### 3.5. PET/CT Findings

Four patients underwent PET/CT examination, and their lesions were observed to be hypermetabolic with increased glucose uptake. Solid pulmonary nodules were noticed in three patients, and abnormal masses were found in the pericardium in one patient with intensive glucose uptake, which confirmed their metastatic property.

### 3.6. Pathological Results

Primary tumor specimens were collected through radical resection (six patients) and thoracotomy biopsy (two patients). Macroscopically, tumors were incanous or taupe in color, with a taupe solid section (some with cystic degeneration), and generally soft in texture. Four patients underwent transthoracic needle biopsy of pulmonary nodules; the biopsy findings confirmed metastases from primary cardiac angiosarcoma in the specimens, presenting as pinkish-grey or dark red. Tumor cells were aligned in a spindle or solid manner, and vascular cavity-like structures were observed in all patients. The differentiation degree of lesions was analyzed among eight patients within our hospital but was not available for the remaining four from other institutions. Immunoreactivity for CD31 and CD34 was most diffuse in all cases, followed by Ki-67, Vimentin and smooth-muscle actin (SMA). The differentiation degree of lesions and Ki-67 index are listed in Table 2.

## 4. Discussion

To the best of our knowledge, this is the largest cohort study of primary cardiac angiosarcoma that performed a multimodality analysis of imaging features. Angiosarcoma is a rare entity, and its etiology is still not fully understood. Definite risk factors include chronic lymphoedema, radiation exposure, and a history of exposure to environmental carcinogens [12]. Several familial reports of cardiac angiosarcoma have indicated its genetic association [13,14]. In our study, the clinical features of all patients and the site and size of all lesions are in correspondence with prior reports.

No studies have thus far reported the role of carcinoembryonic antigens in the pathogenesis of primary cardiac angiosarcoma. CA-125 is a product of the normal endometrium and decidua and is present in the normal ovarian epithelia and epithelia of the colon, lung, pancreas, kidney, and gall bladder; CA-125 is a marker of coelomic epithelial stimulation [15]. Histologically, cardiac angiosarcomas consist of endothelium-lined channels and vascular channels mixed with solid areas of epithelioid and spindle cells, which can be divided into the following three types according to the amount of these three components: a vascular area with anatomizing channels, a solid high-grade epithelioid area, and a spindle cell Kaposi-like area [6]. Because three patients in our study with an increased serum CA-125 level did not have additional diseases, we suppose that serum CA-125 level increases when the epithelioid cells of tumors reach a certain proportion. Thus, serum CA-125 may be a helpful biomarker to monitor cardiac angiosarcoma when other relevant diseases are excluded, especially for men.

Standard treatment is yet to be determined owing to the rarity of this particular disease. Surgical resection is the conventional and preferable choice when possible, and the exact benefits of radiotherapy and chemotherapy remain controversial [6]. In our study, PET/CT was re-performed eight months after chemotherapy with paclitaxel for one patient, which revealed an obvious decrease in tumor size, indicating the effectiveness of chemotherapy for some patients in the late period.

Transthoracic echocardiography is the first-line modality used to identify cardiac masses, but it is dependent on the operator and acoustic windows [16]. In our study, transthoracic echocardiography could not show the lesion even when performed by different operators because of a large amount of pericardial effusion in one patient. Thus, CT and MRI are necessary for further differential diagnosis.

CT is more efficient at tissue characterization and can monitor systemic metastases, especially in the lungs [6,12]. Primary cardiac angiosarcoma often presents as homogeneous or inhomogeneous density on unenhanced CT scans and heterogeneous centripetal enhancement on enhanced images. Yu et al. argued that this phenomenon can be attributed to the lack of vascularity in the mesenchyma of angiosarcoma and abundant vascularity in the parenchyma [8], but they did not discuss this in detail. Microscopically, the mesenchyma of angiosarcoma are composed of epithelial and spindle cells, whereas the parenchyma consists of endothelium-lined vascular channels [6]. In a study of hepatic angiosarcoma reported by Yi et al. [17], highly differentiated hepatic angiosarcoma exhibited annular enhancement in the arterial phase, no internal enhancement, and continuous centripetal filling during the venous and delay phases; however, for poorly differentiated tumors, the enhancement was not obvious or was heterogeneous for all scan phases. In our study, the differentiation degree analysis of lesions with both unenhanced and enhanced-contrast CT scans was only available in three patients. Surprisingly, two lesions with low differentiation showed strong or moderate enhancement in the arterial phase, while the one with high differentiation exhibited mild enhancement in the arterial phase. The Ki-67 index too does not correlate with the differentiation degree of the tumors. This phenomenon may be ascribed to the different proportion of parenchyma in the lesion, and it indicates that the enhancement pattern of tumors on CT scan is not reliable for prediction of the degree of malignancy of primary cardiac angiosarcoma. Studies with large samples are needed to clarify the reason for the inconsistency of this phenomenon between primary hepatic and cardiac angiosarcoma. Low-density areas within lesions showed no enhancement, which may represent hemorrhage or necrosis within the tumor. CT imaging can also provide insights into detailed peripheral infiltration, including pericardial thickening and effusion, the scope of tumor invasion, and the relationship between the lesion and RCA [18]. Small vessels in the mass deriving from the RCA demonstrated that the blood supply of the tumor was from the RCA [9,19].

CT has its limitations. Its histological resolution is poorer than that of MRI. In two patients, only a slightly enlarged right atrium was observed on unenhanced CT scans, which may be neglected by unexperienced doctors. Furthermore, because a contrast agent is usually injected through the right cubital vein, the blood flow artifact in the right atrium can affect the observation of the lesion.

Cardiac MRI has a high temporal and spatial resolution and provides great noninvasive tissue characterization without radiation exposure. It is helpful for heart function evaluation, surgical preparation, and postoperative follow-up. Consequently, cardiac MRI is recommended as the standard technique for the assessment of cardiac tumors [20]. In our study, the lesions of four patients who underwent MRI examination all showed heterogeneous intensity, with hyperintense hemorrhage foci (known as cauliflower-like appearance) on both T1- and T2-weighted images compared with the myocardium, whereas their corresponding unenhanced CT scans showed no sign of hemorrhage, which is in line with the findings of previous studies [21,22]. The lesion of case 4 (Figure 2) presented as irregular pattern on CT scan but was almost round with clear boundary on MRI images. Other MRI features suggesting cardiac malignancies, such as origin from the right heart chamber, broad based attachment, involvement of >1 chamber, size > 5 cm, and ill-defined margins [21], were noticed in the majority or all patients. Signal void was noticed in one lesion with strong enhancement in the arterial phase on CT scan.

“Sunray appearance” was first observed by Yahata et al. in 1994 [23]. The second relevant report was published by Tüdös et al. in 2017 who discovered that hyperintense lines were enhanced bands of the fibrous tissue, whereas hypointense regions were based on unenhanced hematoma and necrosis [24]. Thereafter, “sunray appearance” was regarded as a typical LGE pattern by many [25]. However, Tüdös et al. questioned this argument and indicated that peripheral rim enhancement with central necrosis or hemorrhage may be more typical [26] because another study reported that the enhancing rim was a reliable feature to distinguish cardiac angiosarcoma from cardiac lymphoma [27]. In our study, “sunray appearance” enhancement was absent, and rim enhancement was the common pattern. Additional studies with large samples are needed to determine the typical contrast-enhancement pattern of primary cardiac angiosarcoma on MRI images.

Comparison among studies of imaging features (including CT and MRI) of primary cardiac angiosarcoma and our study is exhibited in Table 4. Our study adds more information in terms of both cardiac CT and MRI imaging features of primary cardiac angiosarcoma when compared to these studies, of which most are isolated case reports.

## 5. Conclusions

Primary cardiac angiosarcoma is rare, and its prognosis is extremely poor. The right atrium is the most common involved site, whereas peripheral invasion, including the pericardium, right coronary artery, tricuspid valve, and right ventricle, was universal. CT features included typical inhomogeneous density on unenhanced scans and heterogeneous centripetal enhancement on enhanced scans. The enhancement pattern shows no exact correlation with the differentiation degree of the tumor. Multiple intrapulmonary solid nodules with halo sign may support the diagnosis. Cauliflower-like appearance on both T1- and T2-weighted MRI is common. The characteristic enhancement pattern of MRI remains to be determined, whereas rim enhancement as a supportive pattern may be considered a diagnostic marker. Familiarity with these imaging characteristics associated with primary cardiac angiosarcoma may assist accurate diagnosis and improve patient prognosis.

## Figures and Tables

**Table 1:** Clinical and imaging features of primary cardiac angiosarcomas.

Case	Age ^a^	Sex	Serum CA-125 (U/mL)	Attachment	Maximum Size (mm) ^b^	Adjacent Invasion ^c^	Metastases	Treatment	Surviving (mo)
1	23	F	Not Known	Lateral wall	80.0	AP RCA	None	Surgery	6(DOD)
2	74	M	Normal	Lateral wall	51.4	AP RCA RV	Lung Lymph nodes	Surgery	7(DOOD)
3	47	F	37.9	Lateral wall	93.0	Limited	None	Surgery	15(AFOD)
4	34	M	Not Known	Lateral wall	66.1	RCA	None	Surgery	Not Known
5	50	M	Not Known	Lateral wall	69.7	AP	None	Surgery	Not Known
6	62	M	99.0	Lateral wall	66.4	AP RCA	Pericardium Lung	Surgery	Not Known
7	61	M	Not Known	Atrial septum	54.4	AP SVC	Lung	Thoracotomy	1(DOD)
8	17	M	Not Known	Lateral wall	47.4	AP	Lung	Thoracotomy, Chemotherapy	7(AWD)
9	26	M	Normal	Lateral wall	79.5	AP RCA RV	Lung	Chemotherapy	9(AWD)
10	61	M	Not Known	Lateral wall	38.4	AP RCA	Lung	Chemotherapy	6(DOD)
11	31	M	Not Known	Lateral wall	81.2	AP RCA	Pericardium Lung Lymph nodes	None	3(AWD)
12	52	M	1339.0	Posterior wall	50.0	AP IVC	Pericardium Lung Brain Kidney Spleen	None	3(AWD)

Abbreviations: F-female; M-male; AP-adjacent pericardium; RCA-right coronary artery; RV-right ventricular; IVC-inferior vena cava; SVC-superior vena cava; DOD-died of disease; DOOD-died of other disease; AFOD-alive free of disease; AWD-alive with disease. ^a^ Mean, 45 ± 18 years. ^b^ Mean, 64.8 ± 16.6 mm. ^c^ Limited adjacent invasion indicates that the tumor is restricted to the right atrium without adjacent invasion.

**Table 2:** CT features of primary cardiac angiosarcoma.

Case	Scan Type	Density	Enhancement Pattern	Differentiation (Ki-67 Index)	Lung Metastasis	GGO Peripheries
1	E	-	Heterogeneous	Moderate (>50%)	N	-
2	P + E	Uneven	Strong Heterogeneous Centripetal	Low (80%)	Y	N
3	P	Uneven	-	Low (85%)	N	-
4	E	-	Heterogeneous Centripetal	Moderate (30%)	N	-
5	P + E	Uneven	Moderate Heterogeneous Centripetal	Low (30%)	N	-
6	E	-	Heterogeneous Centripetal	Moderate (25%+)	Y	N
7	P	Uneven	-	High (10%+)	Y	N
8	P + E	Even	Strong Heterogeneous Centripetal	NK (30%)	Y	Y
9	P + E	Uneven	Strong Heterogeneous Centripetal	NK (50%+)	Y	Y
10	P + E	Even	Mild Heterogeneous Centripetal	High (50%+)	Y *	Y
11	P + E	Uneven	Strong Heterogeneous	NK (50%+)	Y *	Y
12	E	-	Heterogeneous Centripetal	NK (20–25%+)	Y	Y

Abbreviations: GGO-ground-glass opacity, P-CT plain scan; E-CT enhanced scan; NK-Not Known; Y-Yes; N-No * Diffuse smooth thickening interlobular septa in both lower lobes of lungs.

**Table 3:** Magnetic resonance imaging (MRI) features of primary cardiac angiosarcoma.

Case	Boundary	Intensity on T1WI	Intensity on T2WI	Signal Void	Enhancement Pattern	Differentiation	Pericardium Enhancement
2	Regular	Uneven high	Uneven high	Y	Heterogeneous, patchy	Low	N
4	Regular	Uneven high	Uneven high	N	Heterogeneous, rim	Moderate	Y
7	Irregular	Uneven high	Uneven high	N	Heterogeneous, rim	High	N
11	Irregular	Uneven high	Uneven high	N	Heterogeneous, rim	NK	N

Abbreviations: NK-Not Known; Y-Yes; N-No.

**Table 4:** Comparison among studies on imaging features of primary cardiac angiosarcoma.

Study	Number of Patients	CT Features	MRI Features
Antonuzzo et al. [4]	1	None	A mass (53 mm) extending from the free wall of the RA to the anterior mediastinum
Yu et al. [8]	9	Masses in the RA invading pericardium, RV, SVC, and tricuspid valve, presented as homogeneous or inhomogeneous on plain CT scans, and most showed inhomogeneous centripetal enhancement on enhanced CT scans. Pulmonary metastases with GGO peripheries	None
Rao et al. [9]	1	A mass in the RA and RV extending into the pericardium	A large heterogeneous mass in the RA and RV extending into the myocardium.
Yahata et al. [23]	1	None	An extensive mass in pericardial cavity protruding into the RA of heterogeneous signal intensity on T1WI and T2WI, “sunray appearance” enhancement was seen on LGE images.
Tüdös et al. [24]	1	None	A cauliflower mass protruding to the RA with extension toward RV, right appendage, and diffuse obliteration of pericardial cavity around the ventricles, RV motion was severely impaired. Signaling of tumor tissue in T1WI and T2WI was inhomogeneous, mostly isointense to slightly hyperintense. Pattern of enhancement previously described as “sunray appearance” was seen both on T1WI and LGE images, especially LGE images.
Lindsey et al. [25]	1	None	A well-circumscribed, hyperemic mass (27 × 18 mm) located at the supero-posterior wall of the RA and possibly extending from the IVC, presented as hyperintensity on T2WI and LGE. Using first-pass perfusion, the mass did not show contrast as it transited the right side, but, as contrast passed through the coronary arteries, the contrast filled the mass.
Colin et al. [27]	7	None	Tumors located in the right atrial wall and atrial appendage extending to the pericardium, right atrial cavity, and right coronary artery with central necrosis and possibly pulmonary metastasis, and with strong rim enhancement on LGE.
Sun et al. [28]	1	A large mass about 8 × 6 × 5 cm^3^ occupying the RA, SVC and IVC with pericardial and bilateral pleural effusion, with heterogeneous enhancement.	None
Park et al. [29]	1	Multiple-septated fluid collection in right pericardial space.	Diffuse nodular enhancing mass involving the right atrium.
Qian et al. [30]	1	A multifocal high-density shadow and lymph node enlargement (including the right upper lobe) with bilateral pleural effusions, a pericardial effusion and an enlargement of the cardiac area. Pulmonary artery computed tomography angiography (CTA) demonstrated a micro-embolism in the right upper lobe of the lung.	None
Our study	12	Masses in the right atrium invading pericardium, RCA, RV, SVC and IVC, presented as homogeneous or inhomogeneous on unenhanced CT scans and heterogeneous centripetal enhancement on enhanced CT scans, The enhancement pattern shows no exact correlation with the differentiation degree of the tumor. Pulmonary metastases with halo sign was common.	Masses in the right atrium invading pericardium, RCA, RV, SVC and IVC, presented as cauliflower-like appearance on T1WI and T2WI, signal void was noticed in a lesion with strong enhancement for arterial phase on CT scan. RA and RV motion was severely impaired. Rim enhancement was noticed in 3 patients and patchy enhancement was noticed in one patient.

Abbreviations: RA-right atrium; RV-right ventricle; SVC-superior vena cava; IVC-inferior vena cava; RCA-right coronary artery, LGE-Late gadolinium enhancement, GGO-ground-glass opacity.
